# Fatty liver index for hyperuricemia diagnosis: a community-based cohort study

**DOI:** 10.1186/s12902-022-01030-6

**Published:** 2022-04-30

**Authors:** Jianchang Qu, Jingtao Dou, Anping Wang, Yingshu Liu, Lu Lin, Kang Chen, Li Zang, Yiming Mu

**Affiliations:** 1 Medical School of Chinese PLA, Beijing, China; 2 Department of Endocrinology, The First Medical Center, Chinese PLA General Hospital, Beijing, 100853 China; 3 Department of Endocrinology, The 305 Hospital of PLA, Beijing, China

**Keywords:** Hyperuricemia, Fatty liver index, Uric acid, Metabolic score for insulin resistance, Community

## Abstract

**Background:**

Studies have demonstrated the relationship between the fatty liver index (FLI) and metabolism, while few research reported its relationship with hyperuricemia (HUA). This study aimed to predict HUA by determining the relationship between the baseline FLI and HUA events and by validating the FLI–HUA correlation through follow-up.

**Methods:**

This study was a community-based cohort study involving 8851 adults in China. We performed anthropometric assessments and analyzed baseline and follow-up blood samples. HUA was defined as a uric acid level of > 420 µmol/L (7 mg/dL).

**Results:**

Patients with HUA had a higher prevalence of diabetes mellitus, lipid metabolism disorders, and hypertension and higher FLI values than those with normal uric acid levels (*P* < 0.001). Serum uric acid was positively correlated with the FLI (*r* = 0.41, *P* < 0.001); the diagnostic cut-off value of FLI for the diagnosis of HUA was 27.15, with a specificity of 70.9% and sensitivity of 79.6%. FLI was an independent risk factor for HUA, with a 1.72-, 2.74-, and 4.80-fold increase in the risk of developing HUA with increasing FLI quartile levels compared with the FLI at quartile level 1 (*P* < 0.001). After a mean follow-up of 4 years, as the FLI values increased compared with the FLI at quartile level 1, the risk of new-onset HUA increased by 3.10-, 4.89-, and 6.97-fold (*P* < 0.001).

**Conclusion:**

There is a higher incidence of metabolic abnormalities in HUA populations, and FLI is an independent factor that may contribute to HUA development. Therefore, FLI is a potential tool to predict the risk of developing HUA.

## Background

Uric acid is the end product of purine metabolism, and hyperuricemia (HUA) occurs when there is increased uric acid production or decreased uric acid excretion [1]. HUA is associated with metabolic syndrome and its components, including hypertension, diabetes mellitus, and abnormal lipid metabolism [2]. In a study conducted in China, it was reported that HUA can increase the risk of cardiovascular disease [3]. Accordingly, adequate uric acid control may be a factor in managing cardiovascular diseases and metabolic disorders [4]. HUA is easily overlooked in the early stages due to the lack of symptoms, before gouty arthritis and renal tophi develop [5]. Early detection of people at risk for HUA and targeted management are pivotal to reducing the effects of HUA on the body.

In 2006, Bedogni et al. developed the fatty liver index (FLI) for the early prediction of fatty liver disease in the general population [6]. In addition to the definite predictive role of the FLI on fatty liver disease, several studies have suggested that the FLI correlates to the development of metabolic syndrome and its components and even has an early warning effect [7–10] on cardiovascular disease.

FLI and uric acid have an established relationship with metabolic syndrome. However, there are still relatively few reports on the relationship between the FLI and HUA and whether FLI can predict the future risk of developing HUA. Therefore, this study aimed to understand the predictive value of the FLI for HUA by analyzing a large sample from a community-based population in an epidemiological survey.

## Methods

### Study design

In the "Epidemiological follow-up study of tumor risk in Chinese patients with type 2 diabetes mellitus" conducted between April and September 2015, whole population sampling was used. The study was conducted among 10,277 participants aged 18–93 years living in two communities in Beijing, namely Gucheng and Pingguoyuan.

In this study, the inclusion criteria included those aged over 40 years, with complete data sets, and who were able to participate in the follow-up on time. The exclusion criteria included those with pancreatic disease, bile duct disease, hepatitis, malignant tumor, and severe hepatic and renal insufficiency.

A total of 8851 participants had complete data sets, including 3120 men and 5731 women aged 40–91 years, with a mean age ± standard deviation (SD) of 59.95 ± 7.68 years. According to diagnoses, there were 869 cases of HUA, accounting for 9.81% of the total enrolled population, including 582 in men (18.65% of the male population) and 287 in women (5.01% of the female population).

**Fig. 1:**
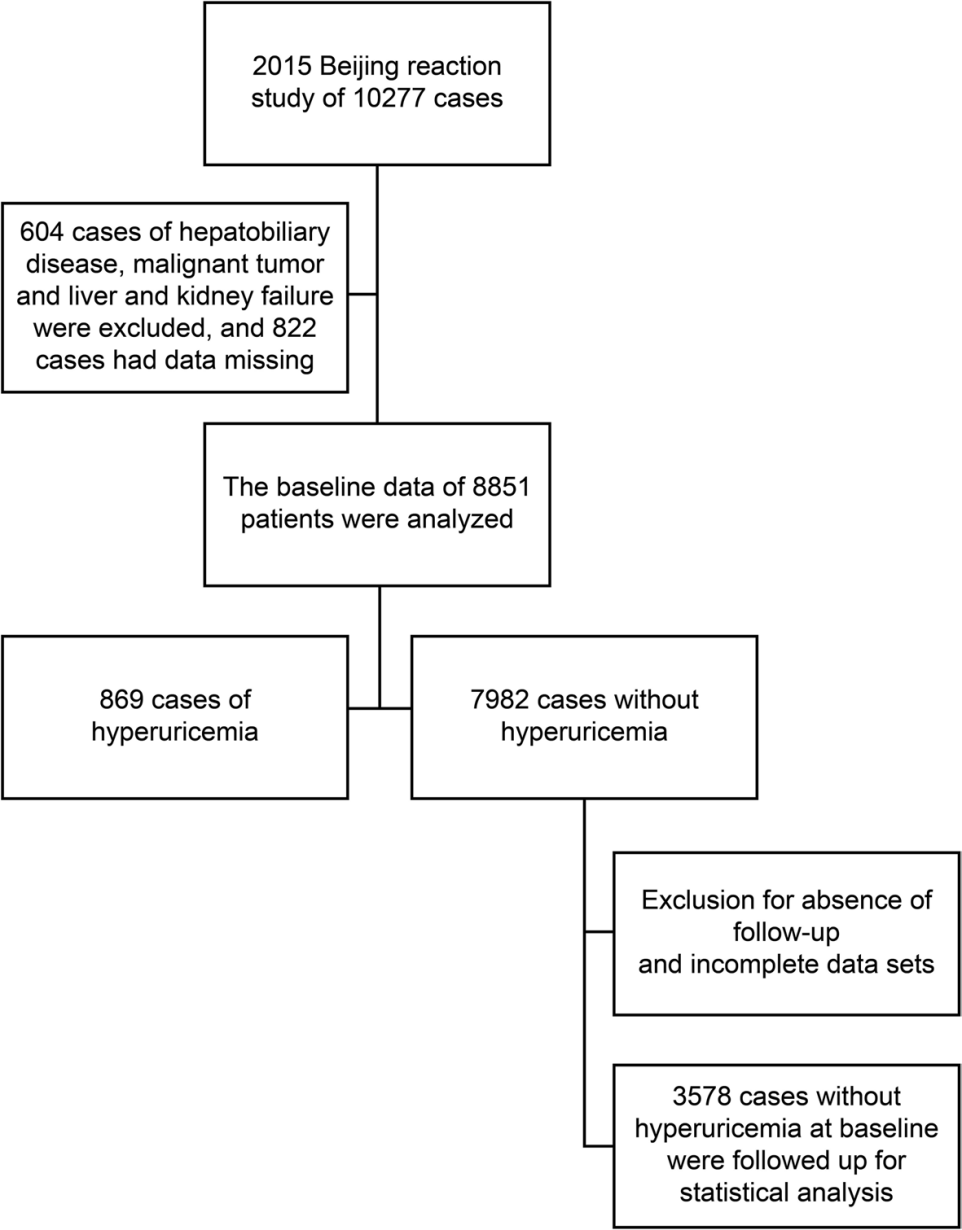
Flow diagram of the enrollment process in the study

Participants were divided by FLI quartile levels as follows: Q1 with 2237 participants (FLI < 14.64), Q2 with 1681 participants (FLI of 14.64–29.38), Q3 with 2737 participants (FLI of 29.39 − 51.42), and Q4 with 2196 (FLI ≥ 51.43) [9, 11]. Between 2019 and 2020, these participants were followed up again. Although there was a decrease in participation, as compared to baseline numbers, due to coronavirus disease 2019 (COVID-19), a total of 3924 participants had complete data sets. In the 2015 HUA-free population, a total of 3578 participants were followed up (Fig. 1), and the occurrence of HUA in this population at the time of 2019 follow-up was calculated. All patients provided written informed consent, and this study protocol was performed in accordance with the Declaration of Helsinki reviewed and was approved by the Ethics Committee of People’s Liberation Army (PLA) General Hospital.

### Demographic and anthropometric assessment

This study was a longitudinal cohort study, with field surveys conducted by professionally trained staff at community health centers using a standardized questionnaire. The questionnaire contained demographic information, including chronic diseases (cardiovascular disease; hypertension; and abnormal glucose, lipid or purine metabolism); alcohol consumption; smoking; long-term medication use; and family history. Height, weight, waist circumference, hip circumference, and blood pressure were measured, and echocardiography, carotid ultrasonography, and electrocardiography were performed at the same time. Trained staff entered the relevant data.

### Biochemical assessment

Following an overnight fast (> 8 h), 10 mL of fasting venous blood was collected from all patients, and sent to the PLA General Hospital to detect biochemical markers, including liver and kidney function, and glucose and lipid metabolism. Patients without diabetes mellitus underwent a 2-h post-load (75 g) glucose test, and fasting blood glucose (FPG) was measured using the glucose oxidase assay. Glycated hemoglobin A1c was measured by high performance liquid chromatography (Bio-Rad). Biochemical markers including liver and kidney function, blood lipids and uric acid, and glutamyl transferase, were measured using the Hitachi 7600–020 automatic biochemical analyzer (Hitachi, Tokyo, Japan).

In this study, we defined HUA as a fasting blood uric acid level of ≥ 420 µmol/L (7 mg/dL), as defined in Chinese Society of Endocrinology’s 2019 Guidelines for the Treatment of Hyperuricemia and Gout [12]. FLI is calculated as follows: FLI = (e 0.953 × log_e_ (triglycerides [TGs]) + 0.139 × body mass index (BMI) + 0.718 × log_e_ (gamma-glutamyl transpeptidase [GGT]) + 0.053 × waist circumference—15.745)/(1 + e 0.953 × log_e_ (TG) + 0.139 × BMI + 0.718 × log_e_ (GGT) + 0.053 × waist circumference—15.745) × 100 [6]. The metabolic score for insulin resistance (METS-IR) was calculated as follows: METS-IR = Ln((2 × FPG) + TG) × BMI)/(Ln(HDL-c)) [13].

### Statistical analysis

Statistical analyses were performed using SPSS 21.0 software (IBM Corp., Armonk, NY, USA). Continuous numerical data were expressed as means ± SD for normally distributed data and as medians (interquartile ranges) for non-normally distributed data. One-way analysis of variance (ANOVA) was used for multiple continuous variables. Count data were expressed as percentages (%), and the chi-square test was used to compare categorical data between groups. A multivariate binary logistic regression model was used to analyze the risk factors of HUA. Two-tailed probability value of *P* < 0.05 was considered as statistically significant.

## Results

### Baseline patient characteristics

Baseline data indicated that age, BMI, systolic blood pressure (SBP), diastolic blood pressure (DBP), GGT, TG, total cholesterol, FPG, serum creatinine (Cr), FLI, and METS-IR were higher in the HUA group than in the non-HUA group, whereas high-density lipoprotein cholesterol (HDL-C) was significantly lower (*P* < 0.001). The prevalence of diabetes mellitus, hypertension, hypertriglyceridemia, low high-density lipoprotein cholesterol levels, and metabolic syndrome were higher in the HUA group than in the non-HUA group (*P* < 0.001) (Table 1).

**Table 1:** Baseline patient characteristics

Parameters	HUA (*n* = 869)	Non-HUA (*n* = 7982)	Statistic	*P*-value
Age (years)	60.96 ± 8.31	59.84 ± 7.61	4.22	< 0.001
BMI (kg/m^2^)	26.88 ± 3.47	25.31 ± 3.51	12.88	< 0.001
WC (cm)	91.3 ± 9.01	85.34 ± 9.34	18.56	< 0.001
SBP (mmHg)	135.76 ± 18.06	132.63 ± 18.49	4.91	< 0.001
DBP (mmHg)	79.41 ± 10.93	78.03 ± 10.83	3.67	< 0.001
GGT (U/L)	27.56 (19.70, 42.33)	19.90 (15.00, 28.50)	17.34	< 0.001
Cr (µmol/L)	83.29 ± 22.71	68.04 ± 14.68	28.21	< 0.001
TC (mmol/L)	4.75 ± 0.99	4.89 ± 0.95	-4.28	< 0.001
TG (mmol/L)	2.00 ± 1.45	1.56 ± 1.07	11.56	< 0.001
HDL-C (mmol/L)	1.29 ± 0.33	1.45 ± 0.38	-12.93	< 0.001
LDL-C (mmol/L)	2.98 ± 0.86	3.11 ± 0.82	-4.64	< 0.001
FPG (mmol/L)	6.09 ± 1.64	5.81 ± 1.69	4.78	< 0.001
FLI	49.45 (30.51, 71.49)	27.23 (13.51, 48.35)	19.84	< 0.001
Sex (M/F)	582/287	2508/5474	460.01	< 0.001
Smoking, *n* (%)	266 (30.6)	1332 (16.7)	99.94	< 0.001
Drinking, *n* (%)	423 (48.7)	2112 (26.5)	182.22	< 0.001
HTN, *n* (%)	643 (73.4)	4422 (55.4)	99.41	< 0.001
DM, *n* (%)	366 (42.1)	2304 (28.9)	62.30	< 0.001
High TG, *n* (%)	436 (50.2)	2566 (32.1)	108.13	< 0.001
Low HDL, *n* (%)	211 (24.3)	909 (11.4)	115.54	< 0.001
MS, *n* (%)	421 (48.5)	2105 (26.4)	191.52	< 0.001
METS-IR	41.37 ± 7.26	37.19 ± 13.28	9.47	< 0.001

Data are expressed as mean ± standard deviation for normally distributed variables and as median (interquartile range) for non-normally distributed variables

*BMI* body mass index, *WC* waist circumference, *SBP* systolic blood pressure, *DBP* diastolic blood pressure, *GGT* glutamyl transpeptidase, *CR* creatinine, *TC* total cholesterol, *TG* triglyceride, *HDL-C* high-density lipoprotein cholesterol, *LDL-C* low-density lipoprotein cholesterol, *FPG* fasting plasma glucose, *FLI* fatty liver index, *HTN* hypertension, *DM* diabetes mellitus, *MS* metabolic syndrome, *METS-IR* metabolic score for insulin resistance

### Comparison of baseline characteristics at the different FLI quartile levels

Differences in several biochemical markers were compared among four groups (Q1, Q2, Q3, and Q4). BMI, SBP, DBP, TG, FPG, Cr, uric acid, and METS-IR were found to be significantly increased (*P* < 0.001), whereas HDL-C gradually decreased (*P* < 0.001) as the FLI quartile levels increased (Table 2).

**Table 2:** Comparison of baseline characteristics at different FLI quartile levels

	Q1 (*n* = 2237)	Q2 (*n* = 1681)	Q3 (*n* = 2737)	Q4 (*n* = 2196)	Statistical quantity	*P*-value
Age (years)	59.26 ± 8.01	60.23 ± 7.75^a^	60.54 ± 7.54^a^	59.71 ± 7.43^abc^	13.80	< 0.001
BMI (kg/m^2^)	21.9 ± 2.03	24.3 ± 1.8^a^	26.12 ± 2.1^ab^	29.11 ± 3.24^abc^	3835.46	< 0.001
WC (cm)	75.78 ± 5.92	83.04 ± 5^a^	87.96 ± 5.51^ab^	95.76 ± 7.49^abc^	4493.54	< 0.001
SBP (mmHg)	125.95 ± 17.94	131.92 ± 18.12^a^	135.12 ± 17.88^ab^	138.01 ± 17.78^abc^	198.87	< 0.001
DBP (mmHg)	74.79 ± 10.14	77.29 ± 10.27^a^	78.94 ± 10.58^ab^	81.26 ± 11.23^abc^	156.81	< 0.001
GGT (U/L)	14.80 (12.1,18.3)	18.00 (14.4, 23.12)^a^	22.50 (17.6, 29.7)^ab^	31.5 (23.2, 47.4)^abc^	406.19	< 0.001
CR (µmol/L)	66.32 ± 14	68.63 ± 14.01^a^	70.34 ± 16.5^ab^	72.47 ± 18.97^abc^	61.45	< 0.001
TC (mmol/L)	4.79 ± 0.89	4.8 ± 0.91	4.87 ± 0.96^ab^	5.01 ± 1.02^abc^	25.62	< 0.001
TG (mmol/L)	0.98 ± 0.37	1.28 ± 0.5^a^	1.61 ± 0.71^ab^	2.46 ± 1.69^abc^	990.99	< 0.001
HDL (mmol/L)	1.7 ± 0.39	1.47 ± 0.32^a^	1.36 ± 0.32^ab^	1.23 ± 0.3^abc^	846.84	< 0.001
LDL (mmol/L)	2.97 ± 0.76	3.09 ± 0.8^a^	3.16 ± 0.82^ab^	3.16 ± 0.88^ab^	29.47	< 0.001
UA (µmol/L)	262.19 ± 60.98	288.9 ± 64.26^a^	310.58 ± 70.68^ab^	341.71 ± 79.22^abc^	550.72	< 0.001
FPG (mmol/L)	5.46 ± 1.46	5.66 ± 1.55^a^	5.91 ± 1.67^ab^	6.26 ± 1.9^abc^	99.18	< 0.001
HUA, *n* (%)	72 (3.2)	105 (6.2)^a^	305 (11.1)^ab^	448 (20.4)^abc^	378.01	< 0.001
HTN, *n* (%)	850 (38.0)	876 (52.1)^a^	1694 (61.9)^ab^	1645 (74.9)^abc^	577.62	< 0.001
DM, *n* (%)	425 (19.0)	441 (26.2)^a^	876 (32.0)^ab^	928 (42.3)^abc^	280.74	< 0.001
H-TG, *n* (%)	91 (4.1)	282 (16.8)^a^	1055 (38.5)^ab^	1574 (71.7)^abc^	2413.92	< 0.001
L-HDL, *n* (%)	49 (2.2)	106 (5.9)^a^	377 (13.8)^ab^	588 (26.8)^abc^	664.65	< 0.001
MS, *n* (%)	12 (0.5)	123 (6.3)^a^	843 (30.8)^ab^	1554 (70.7)^abc^	3035.19	< 0.001
METS-IR	29.67 ± 3.51	34.68 ± 3.00^a^	38.77 ± 3.68^ab^	46.30 ± 21.91^abc^	893.90	< 0.001

^a^*P* < 0.05 compared with Q1

^b^*P* < 0.05 compared with Q2

^c^*P* < 0.05 compared with Q3

Data are expressed as mean ± standard deviation for normally distributed variables and as median (interquartile range) for non-normally distributed variables

BMI, body mass index; WC, waist circumference; SBP, systolic blood pressure; DBP, diastolic blood pressure; GGT, glutamyl transpeptidase; CR, creatinine; TC, total cholesterol; TG, triglyceride; HDL-C, high-density lipoprotein cholesterol; LDL-C, low-density lipoprotein cholesterol; FPG, fasting plasma glucose; FLI, fatty liver index, HTN, hypertension; DM, diabetes mellitus; MS, metabolic syndrome; METS-IR, metabolic score for insulin resistance

### Comparison of the correlations between serum uric acid, the FLI, and the METS-IR

**Fig. 2:**
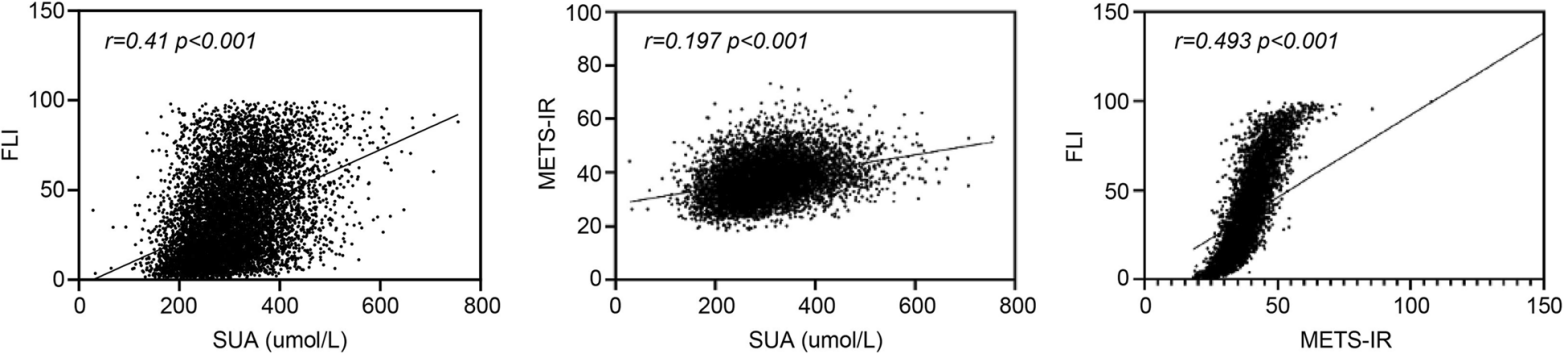
Comparison of correlations between SUA, FLI, and METS-IR. SUA, serum uric acid; FLI, fatty liver index; METS-IR, metabolic score for insulin resistance

The Spearman and Pearson correlations were used to analyze the correlation between serum uric acid and the FLI. Serum uric acid was significantly correlated with FLI and METS-IR (*P* < 0.001), and the FLI was significantly correlated with METS-IR (*P* < 0.001) (Fig. 2).

### Diagnostic efficacy of the FLI for HUA

**Fig. 3:**
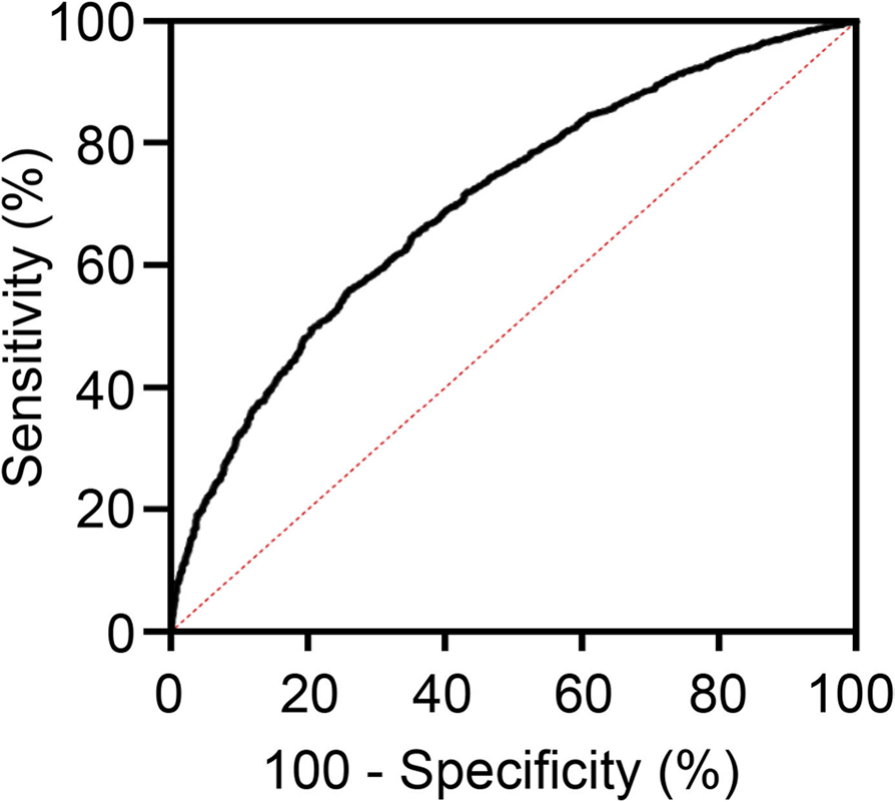
Diagnostic efficacy of the fatty liver index for HUA. HUA, hyperuricemia

The receiver operating characteristic (ROC) curve suggested that the diagnostic cut-off value of FLI for the diagnosis of HUA was 27.15, with a specificity of 70.9% and sensitivity of 79.6% (area under the ROC curve 0.703, *P* < 0.001) (Fig. 3).

### Analysis of the factors influencing HUA

**Fig. 4:**
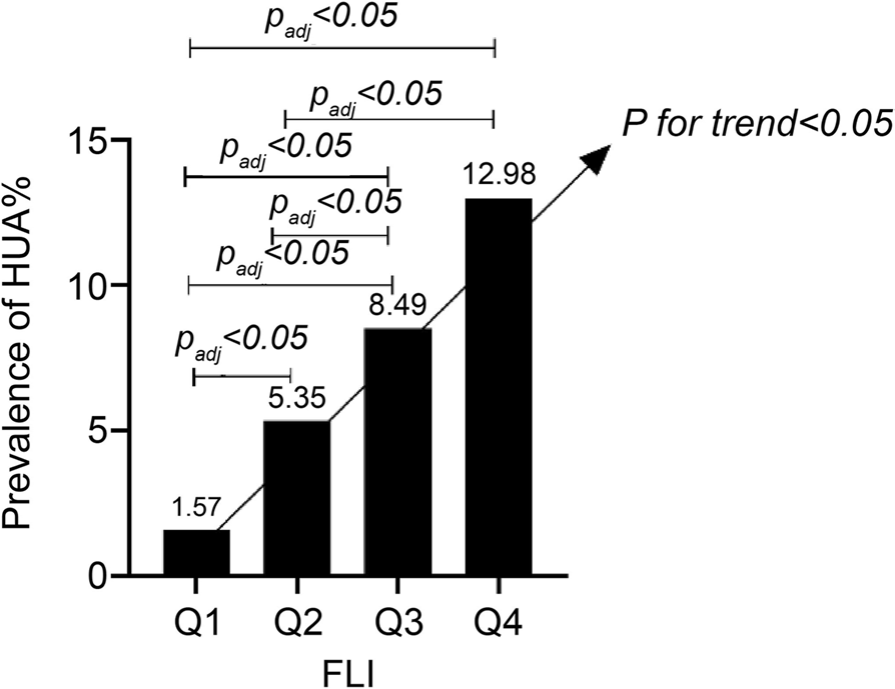
Prevalence of HUA per FLI quartile. FLI, fatty liver index; HUA, hyperuricemia

Using the occurrence of HUA as the dependent variable, the results of binary multivariate logistic regression analysis with gradual adjustments for confounders suggested that the risk of HUA also varied according to the FLI quartile levels, with a 1.72-, 2.74-, and 4.80-fold increase in the risk of HUA, compared with Q1 (*P* < 0.001) (Table 3, Fig. 4).

**Table 3:** Influence of fatty liver index quartile levels on hyperuricemia by binary multivariate logistic regression analysis

		B	S.E	Wals	*P*-value	OR	95% CI	
Model 1	Q1			323.55	< 0.001	1		
	Q2	0.68	0.16	18.94	< 0.001	1.98	1.45	2.68
	Q3	1.31	0.13	94.97	< 0.001	3.70	2.84	4.80
	Q4	2.01	0.13	235.99	< 0.001	7.45	5.76	9.62
Model 2	Q1			241.06	< 0.001	1		
	Q2	0.59	0.16	13.89	< 0.001	1.80	1.32	2.46
	Q3	1.13	0.14	69.63	< 0.001	3.11	2.38	4.05
	Q4	1.77	0.13	177.008	< 0.001	5.85	4.51	7.59
Model 3	Q1			162.973	< 0.001	1		
	Q2	0.54	0.16	11.171	< 0.001	1.72	1.25	2.36
	Q3	1.01	0.14	51.213	< 0.001	2.74	2.08	3.60
	Q4	1.57	0.14	124.508	< 0.001	4.80	3.64	6.32

Model 1: Not adjusted

Model 2: Adjusted for sex, age, smoking, drinking, and physical activity

Model 3: Adjusted for sex, age, smoking, drinking, physical activity, Cr, diabetes, hyperlipidemia, and hypertension

*Cr* creatinine

### Influence of the FLI quartile levels on post-follow-up diagnosis of new-onset HUA

For the HUA-free population at baseline, a mean follow-up of 4 years revealed a progressive trend of increasing HUA prevalence with increasing baseline FLI quartile levels (*P* < 0.001). The prevalence of HUA for each FLI quartile level was 1.57%, 5.35%, 8.49%, and 12.98%, respectively (Fig. 4). Using the occurrence of new-onset HUA as the dependent variable, the results of binary multivariate logistic regression analysis with gradual adjustments for confounders suggested that the risk of new-onset HUA after follow-up also varied across the baseline FLI quartile levels, with a 3.10-, 4.89-, and 6.97-fold increase in the risk of developing HUA, compared with Q1 (*P* < 0.001) (Table 4).

**Table 4:** Influence of fatty liver index quartile levels on newly diagnosed new-onset hyperuricemia by binary multivariate logistic regression analysis

		B	S.E	Wals	*P*-value	OR	95% CI	
Model 1	Q1			76.60	< 0.001	1.00		
	Q2	1.27	0.31	16.94	< 0.001	3.55	1.94	6.48
	Q3	1.76	0.29	35.68	< 0.001	5.82	3.27	10.37
	Q4	2.24	0.29	60.59	< 0.001	9.35	5.33	16.42
Model 2	Q1			57.55	< 0.001			
	Q2	1.17	0.31	14.22	< 0.001	3.21	1.75	5.89
	Q3	1.62	0.30	29.93	< 0.001	5.07	2.84	9.08
	Q4	2.00	0.29	47.26	< 0.001	7.36	4.17	13.00
Model 3	Q1			50.36	< 0.001			
	Q2	1.13	0.31	13.23	< 0.001	3.10	1.68	5.69
	Q3	1.59	0.30	28.01	< 0.001	4.89	2.72	8.80
	Q4	1.94	0.30	42.49	< 0.001	6.97	3.89	12.50

Model 1: Not adjusted

Model 2: Adjusted for sex, age, smoking, drinking, and physical activity

Model 3: Adjusted for sex, age, smoking, drinking, physical activity, Cr, diabetes, hyperlipidemia, and hypertension

*Cr* creatinine

## Discussion

In this study, there is a higher incidence of metabolic abnormalities among community HUA populations. FLI is an independent influencing factor in the development of HUA, and FLI can predict the risk of future HUA.

FLI a good predictor of nonalcoholic fatty liver disease (NAFLD) [14]. In 2018, research proposed to replace NAFLD with metabolic-associated fatty liver disease (MAFLD), highlighting the contribution of metabolic factors in fat deposition in the liver [15]. MAFLD may be accompanied by insulin resistance (IR) and abnormalities in lipid and glucose metabolism, hypertension, HUA, and metabolic syndrome, and these metabolic abnormalities predispose patients to MAFLD. The proposed mechanism is related to the reactive oxygen species/c-Jun N-terminal kinase/activator protein-1 signaling pathway, which causes fat accumulation in the liver [16]. Uric acid is related to hepatocyte destruction in the early stage of NAFLD [17]. Serum uric acid is positively correlated with NAFLD, and elevated serum uric acid levels can be used as an independent predictor of NAFLD [18]. Another study found the higher serum uric acid group had a nearly twofold increased risk of NAFLD compared with the lower group [19].

Uric acid is closely related to metabolic syndrome, and HUA is associated with metabolic syndrome and its components, and also with aortic and cardiac function [20–23]. According to the baseline data of this study, the prevalence of metabolic syndrome in the HUA population was higher than in the non-HUA population, which was similar with the results of Jeong et al. [20]. In another study, it proposed that HUA should be used to predict future metabolic syndrome [24]. Tani et al. [25] suggested that uric acid concentration is a better marker of metabolic syndrome components among women. Multiple metabolic syndrome components can increase the risk of HUA, of which hypertension and low-density lipoprotein cholesterol likely being the most important determinants [26]. Similarly, this study also showed that the prevalence of hypertension, diabetes mellitus, hypoalphalipoproteinemia, and hypertriglyceridemia was significantly higher in the HUA population than in the non-HUA population, fully reflecting the close relationship between uric acid and metabolic syndrome components.

This study also revealed that there was a significant correlation between serum uric acid and FLI quartile levels (*r* = 0.41, *P* < 0.001). The cutoff value of FLI for the diagnosis of HUA was 27.15, with a specificity of 70.9% and sensitivity of 79.6%. In addition, the prevalence of metabolic syndrome gradually increased with increasing FLI quartile levels, and FLI played a significant role in predicting metabolic syndrome. Cheng et al. [27] concluded that FLI could be used for metabolic syndrome identification and is a reliable tool to predict metabolic syndrome with an area under the ROC curve of 0.879. A Japanese study suggested that higher FLI values predicted the risk of new-onset diabetes mellitus [8], and similarly in our study, the prevalence of diabetes mellitus and hypertension gradually increased with increasing FLI quartile levels. In a study in China, it was reported that the FLI was strongly associated with new-onset hypertension, with higher FLI quartile levels at baseline correlating to an increased risk of new-onset hypertension by 2.17- and 3.00-fold for Q3 and Q4, respectively, compared with lower FLI quartile levels [28]. FLI is a good predictor of metabolic syndrome, which is closely related to serum uric acid. Further, the FLI is significantly correlated with serum uric acid. Therefore, it is reasonable to suggest that the FLI may have a unique predictive value for HUA.

After adjusting for confounding factors, the results suggested that the risk of developing HUA also varied according to the FLI quartile levels and that the FLI quartile level was an independent risk factor for developing HUA, with a 1.72-, 2.74-, and 4.80-fold increased risk of developing HUA, respectively, as the FLI quartile levels increased, compared with Q1 (*P* < 0.001). In the HUA-free population at baseline, follow-up was performed after 39 months, and the results suggested that the higher the baseline FLI quartile level, the higher the prevalence of new-onset HUA at follow-up.

In addition to various metabolic factors, this study also considered the relationship of the FLI and serum uric acid concentration and the relationship between HUA and IR. IR and comorbid hyperinsulinemia are strongly associated with the development of HUA; some studies have shown that hyperinsulinemia can lead to HUA and that lowering IR can reduce serum uric acid levels and the risk of gout [29], while it is unlikely that lowering serum uric acid levels will reduce IR, suggesting that this effect is unidirectional. In another study, it was suggested that HUA was an independent influencing factor for IR and diabetes mellitus [30]. Li et al. [31] suggested that severe IR was more likely to develop HUA and hypertension. FLI can predict NAFLD or MAFLD, and the latter is an independent risk factor for IR, while some studies have also suggested that FLI is positively correlated with the homeostasis model assessment of IR index [32]. The baseline data of this study further showed that the METS-IR was significantly higher in the HUA population than in the non-HUA population and that it increased with increasing FLI quartile levels. Uric acid levels were positively correlated with the METS-IR, and FLI showed a significant positive correlation with the METS-IR. In addition, previous study have shown that the METS-IR was positively correlated with IR [7], while those with an elevated METS-IR had a higher risk of developing hypertension [33] and hypertension in turn increases the risk of HUA [26].

This study had some limitations. First, the number of absent follow-ups was large due to COVID-19. Second, the population that we observed was a whole-group sample, and women were overrepresented in this population. Third, there was a lack of a long follow-up period. Lastly, a more direct and objective marker for IR was lacking, as insulin was not measured.

## Conclusions

Community HUA populations have a higher incidence of metabolic abnormalities. FLI is an independent influencing factor in the development of HUA, and the FLI can predict the risk of future HUA. Therefore, adequate attention should be paid to the uric acid biochemical marker.

## Data Availability

The data that support the findings of this study are not publicly available due to their containing information that could compromise the privacy of research participants but are available from the corresponding author OR Data sharing committee upon reasonable request.

## Abbreviations

FLI: Fatty liver index
HUA: Hyperuricemia
SD: Standard deviation
COVID-19: Coronavirus disease 2019
PLA: People’s Liberation Army
FPG: Fasting blood glucose
TGs: Triglycerides
GGT: Gamma-glutamyl transpeptidase
METS-IR: Metabolic score for insulin resistance
SBP: Systolic blood pressure
DBP: Diastolic blood pressure
Cr: Serum creatinine
HDL-C: High-density lipoprotein cholesterol
ROC: Receiver operating characteristic
NAFLD: Nonalcoholic fatty liver disease
MAFLD: Metabolic-associated fatty liver disease
IR: Insulin resistance

## References

[1] Maiuolo J, Oppedisano F, Gratteri S, Muscoli C, Mollace V (2016). Regulation of uric acid metabolism and excretion. Int J Cardiol.

[2] Zhang S, Wang Y, Cheng J, Huangfu N, Zhao R, Xu Z (2019). Hyperuricemia and Cardiovascular Disease. Curr Pharm Des.

[3] Mohammed AQ, Abdu FA, Liu L, Zhang W, Yin G, Xu Y (2021). Hyperuricemia Predicts Adverse Outcomes After Myocardial Infarction With Non-obstructive Coronary Arteries. Front Med.

[4] Zafar U, Ali Z, Naz S, Iftikhar A, Khaliq S, Lone K. Serum uric acid in relation with insulin resistance associated indices in metabolic syndrome and healthy subjects----an observational study. Pakistan Journal of Medical and Health Sciences. 2021;15:816–9.

[5] Huo S, Wang H, Yan M, Xu P, Song T, Li C (2021). Urinary Proteomic Characteristics of Hyperuricemia and Their Possible Links with the Occurrence of Its Concomitant Diseases. ACS Omega.

[6] Bedogni G, Bellentani S, Miglioli L, Masutti F, Passalacqua M, Castiglione A (2006). The Fatty Liver Index: a simple and accurate predictor of hepatic steatosis in the general population. BMC Gastroenterol.

[7] Higashiura Y, Furuhashi M, Tanaka M, Takahashi S, Koyama M, Ohnishi H (2021). High level of fatty liver index predicts new onset of diabetes mellitus during a 10-year period in healthy subjects. Sci Rep.

[8] Olubamwo OO, Virtanen JK, Pihlajamaki J, Tuomainen TP (2019). Association of fatty liver index with risk of incident type 2 diabetes by metabolic syndrome status in an Eastern Finland male cohort: a prospective study. BMJ Open.

[9] Kim JH, Moon JS, Byun SJ, Lee JH, Kang DR, Sung KC (2020). Fatty liver index and development of cardiovascular disease in Koreans without pre-existing myocardial infarction and ischemic stroke: a large population-based study. Cardiovasc Diabetol.

[10] Khang AR, Lee HW, Yi D, Kang YH, Son SM. The fatty liver index, a simple and useful predictor of metabolic syndrome: analysis of the Korea National Health and Nutrition Examination Survey 2010–2011. Diab, Metabol Syndr Obes : Targets Ther. 2019;12:181–90. 10.2147/DMSO.S189544 PMC6353218 30774403

[11] Wang C, Cai Z, Deng X, Li H, Zhao Z, Guo C (2021). Association of Hepatic Steatosis Index and Fatty Liver Index with Carotid Atherosclerosis in Type 2 Diabetes. Int J Med Sci.

[12] Endocrinology CSo (2020). Guidelines for the diagnosis and treatment of hyperuricemia and gout in China (2019). Chin J Endocrinol Metabol.

[13] Bello-Chavolla OY, Almeda-Valdes P, Gomez-Velasco D, Viveros-Ruiz T, Cruz-Bautista I, Romo-Romo A (2018). METS-IR, a novel score to evaluate insulin sensitivity, is predictive of visceral adiposity and incident type 2 diabetes. Eur J Endocrinol.

[14] Hsu CL, Wu FZ, Lin KH, Chen YH, Wu PC, Chen YH (2019). Role of Fatty Liver Index and Metabolic Factors in the Prediction of Nonalcoholic Fatty Liver Disease in a Lean Population Receiving Health Checkup. Clin Transl Gastroenterol.

[15] Eslam M, Sanyal AJ, George J (2020). MAFLD: A Consensus-Driven Proposed Nomenclature for Metabolic Associated Fatty Liver Disease. Gastroenterology.

[16] Xie D, Zhao H, Lu J, He F, Liu W, Yu W (2021). High uric acid induces liver fat accumulation via ROS/JNK/AP-1 signaling. Am J Physiol Endocrinol Metab.

[17] Sertoglu E, Ercin CN, Celebi G, Gurel H, Kayadibi H, Genc H (2014). The relationship of serum uric acid with non-alcoholic fatty liver disease. Clin Biochem.

[18] Wei F, Li J, Chen C, Zhang K, Cao L, Wang X (2020). Higher Serum Uric Acid Level Predicts Non-alcoholic Fatty Liver Disease: A 4-Year Prospective Cohort Study. Front Endocrinol.

[19] Darmawan G, Hamijoyo L, Hasan I (2017). Association between Serum Uric Acid and Non-Alcoholic Fatty Liver Disease: A Meta-Analysis. Acta Med Indones.

[20] Jeong H, Moon JE, Jeon CH (2020). Hyperuricemia is Associated With an Increased Prevalence of Metabolic Syndrome in a General Population and a Decreased Prevalence of Diabetes in Men. J Rheum Dis.

[21] Dobrzyńska M, Przysławski J (2020). The relationship between serum uric acid concentration and cardiovascular risk factors in normotensivepostmenopausal women with dyslipidemia. Acta scientiarum polonorum Technologia alimentaria.

[22] Johnson RJ (2015). Why focus on uric acid?. Curr Med Res Opin.

[23] Ni Q, Lu X, Chen C, Du H, Zhang R (2019). Risk factors for the development of hyperuricemia: A STROBE-compliant cross-sectional and longitudinal study. Medicine.

[24] Liu CW, Chang WC, Lee CC, Chen KH, Wu YW, Hwang JJ (2018). Hyperuricemia Is Associated With a Higher Prevalence of Metabolic Syndrome in Military Individuals. Mil Med.

[25] Tani S, Matsuo R, Imatake K, Suzuki Y, Takahashi A, Yagi T (2020). The serum uric acid level in females may be a better indicator of metabolic syndrome and its components than in males in a Japanese population. J Cardiol.

[26] Liu JH, Ma QH, Xu Y, Chen X, Pan CW. Metabolic Syndrome and 5-Year Incident Hyperuricemia Among Older Chinese Adults: A Community-Based Cohort Study. Diab, Metabol Syndr Obes : Targets Ther. 2020;13:4191–200. 10.2147/DMSO.S278542 PMC7654523 33192081

[27] Cheng YL, Wang YJ, Lan KH, Huo TI, Huang YH, Su CW (2017). Fatty Liver Index and Lipid Accumulation Product Can Predict Metabolic Syndrome in Subjects without Fatty Liver Disease. Gastroenterol Res Pract.

[28] Zhou K, Cen J (2018). The fatty liver index (FLI) and incident hypertension: a longitudinal study among Chinese population. Lipids Health Dis.

[29] McCormick N, O’Connor MJ, Yokose C, Merriman TR, Mount DB, Leong A, et al. Assessing the Causal Relationships Between Insulin Resistance and Hyperuricemia and Gout Using Bidirectional Mendelian Randomization. Arthritis Rheumatol (Hoboken, NJ). 2021;73:2096–104. 10.1002/art.41779 PMC8568618 33982892

[30] Krishnan E, Pandya BJ, Chung L, Hariri A, Dabbous O (2012). Hyperuricemia in young adults and risk of insulin resistance, prediabetes, and diabetes: a 15-year follow-up study. Am J Epidemiol.

[31] Li Y, You A, Tomlinson B, Yue L, Zhao K, Fan H (2021). Insulin resistance surrogates predict hypertension plus hyperuricemia. J Diab Investig.

[32] Wei C, Wang Z, Yang T, Sun M, Endocrinology DO. Study of correlation between fatty liver index and insulin resistance in middle-aged and elderly population in China. J Clin Internal Med. 2019;36:4.

[33] Bello-Chavolla OY, Antonio-Villa NE, Vargas-Vázquez A, Martagón AJ, Mehta R, Arellano-Campos O (2019). Prediction of incident hypertension and arterial stiffness using the non-insulin-based metabolic score for insulin resistance (METS-IR) index. J Clin Hypertens (Greenwich).

